# An Optimized Method to Assess Viable *Escherichia coli* O157:H7 in Agricultural Soil Using Combined Propidium Monoazide Staining and Quantitative PCR

**DOI:** 10.3389/fmicb.2020.01809

**Published:** 2020-07-31

**Authors:** Yulong Fu, Zhe Ye, Yangyang Jia, Jiahui Fan, Muhammad Zaffar Hashmi, Chaofeng Shen

**Affiliations:** ^1^Department of Environmental Engineering, College of Environmental and Resource Sciences, Zhejiang University, Hangzhou, China; ^2^Department of Chemistry, COMSATS University Islamabad, Islamabad, Pakistan; ^3^Zhejiang Provincial Key Laboratory for Water Pollution Control and Environmental Safety, Hangzhou, China

**Keywords:** *E. coli* O157:H7, PMA, qPCR, agricultural soils, viable cells

## Abstract

Agricultural soil contaminated by manure is becoming an important source for the transmission of foodborne pathogens. There is an urgent need for a rapid and accurate method for viable pathogen detection in agricultural soil samples. Propidium monoazide (PMA) is a DNA-binding dye that can inhibit the amplification of DNA from dead cells through subsequent quantitative polymerase chain reaction (qPCR), thus allowing for viable cells detection and quantification. The objective of this study was to detect viable *Escherichia coli* O157:H7 in the agricultural soils by PMA-qPCR. In this study, cell extraction and gradient density centrifugation were incorporated before PMA-qPCR to reduce the interference of soil particle including turbidity and a high ratio of dead cells. The optimized treatment conditions were determined as follows, the maximum removal of DNA from dead cells was achieved by 1.067 g/mL Percoll of centrifugation and 50 μM PMA treatment. Under these conditions, the turbidity of paddy soil suspensions decreased from 3500 to 28.4 nephelometric turbidity units (NTU), and the ratio of viable cells to dead cells increased from 0.001 to 1.025%. For typical agricultural soils collected in China, as low as 10^2^colony-forming units (CFU)/g of viable cells could be accurately detected in the presence of a large number of dead cells (10^7^ CFU/g) by the optimized PMA-qPCR. Significantly, with comparable accuracy, the optimized PMA-qPCR assay was more sensitive, accessible and rapid than conventional culture methods. In addition, the viable but non-culturable (VBNC) state of *E. coli* O157:H7 cells in paddy soils, which often escaped the detection by conventional culture methods, could be quantitatively characterized by the optimized PMA-qPCR method. Potentially, the optimized PMA-qPCR can be further applied for viable pathogens detection and give insight into the prevalence of VBNC *E. coli* O157:H7 in agricultural soil.

## Introduction

Fresh produces have frequently been associated with foodborne disease outbreaks due to *Escherichia coli* O157:H7 (Yeni et al., 2016; Wadamori et al., 2017). *E. coli* O157:H7 poses a significant threat to public health because of its low infectious dose and severe pathogenicity, leading to complications such as hemolytic uremic syndrome (HUS) and thrombotic thrombocytopenic purpura (TTP) (Pennington, 2010). Agricultural soils are not only an important reservoir for pathogens, but also plays an important role in the fecal-oral route (Alegbeleye et al., 2018). To better protect public health, there is a great demand for rapid, sensitive, specific, and accurate quantification of pathogens in agricultural soil.

Traditional methods for pathogen detection in soils rely on culture techniques. However, these methods are time-consuming and labor-intensive. Many pathogens can enter a transient state of dormancy, such as a viable but non-culturable (VBNC) state, which prevents their detection by the culture media (Li L. et al., 2014). To overcome the drawbacks of culture-based methods, quantitative polymerase chain reaction (qPCR) has widely been used for pathogen detection in the environmental samples, due to its rapid, sensitive, and specific characteristics (Warish et al., 2015; Oliver et al., 2016). A major challenge for qPCR is that it is incapable of distinguishing between viable and dead cells, leading to false positive results and overestimation of the concentration of viable pathogens. Propidium monoazide (PMA), a nucleic acid dye, can bind to DNA by intercalation. Under bright light exposure, the azide group from PMA is converted to a highly reactive nitrene, which reacts with the DNA bases to form a stable nitrogen-carbon bond. Crossed-linked DNA strands are not amplified during PCR (Nocker et al., 2006). In addition, viable cells have intact cell membranes that prevent PMA from penetrating into cells, so PMA cannot form covalent bonds with DNA. PMA-qPCR assay has been applied to the detection of viable pathogens including *E. coli*, *Legionella pneumophila*, *Salmonella typhimurium*, and *Staphylococcus aureus* in food and water (Li et al., 2015; Gyawali et al., 2016; Heise et al., 2016; Casanovas-Massana et al., 2018; Yuan et al., 2018; Ge et al., 2019). Recently, PMA method has also been applied in agricultural soil (Gustave et al., 2019). However, the information about pathogens in the agricultural soils detected by PMA-qPCR is still very limited.

There is evidence that PMA has limitations when applied to the complex soil matrices. The effectiveness of PMA treatment should be investigated due to the inactivation effects of turbidity, and a high ratio of dead cells. Moreover, the concentration of PMA is a key methodological parameter that affect PMA efficiency. In detail, matrix turbidity has an effect on the cross-linking efficiency of light activation, leading to false positive results (Silva and Domingues, 2015). It is likely that the agricultural soils can harbor significant amounts of dead cells, and as high as 40% of prokaryotic DNA was extracellular or from cells that were no longer intact in soils (Carini et al., 2017). A high proportion of dead bacteria makes PMA ineffective and leads to false positive results (Fittipaldi et al., 2012; Seinige et al., 2014). In addition, high concentrations of PMA exert toxic effects on viable cells, resulting in false negative results (Liu and Mustapha, 2014). To our knowledge, the optimal conditions of PMA application for viable pathogens detection in soils are still not clear.

The objective of this research was to develop an optimized PMA-qPCR method for detection and quantification of viable *E. coli* O157:H7 in the agricultural soils. In order to reduce PMA limitations, we optimized cell extraction method (Step 1), gradient density centrifugation method (Step 2) and PMA concentrations. To simulate a high ratio of dead cells in soils, we used sterilized soil inoculated with a mixture of viable and dead *E. coli* O157:H7 (viable/dead ratio of 0.001%). The number of viable cells detected by PMA-qPCR were used for evaluating the efficiency of the optimized PMA-qPCR. Additionally, we applied the optimized method to quantify viable *E. coli* O157:H7 cells in the Chinese typical agricultural soils. To our knowledge, the PMA coupled with qPCR assay was, for the first time, applied for quantification of viable cells in the presence of a high concentration of dead cells in the agricultural soils.

## Materials and Methods

### Soil Sampling and Collection

Black soil, red soil, alluvial soil, and paddy soil are representative of the main soil types in China (Li et al., 2013; Teng et al., 2014). The four soils were collected from four agricultural areas across China. Black soil with a silty clay texture was sampled from the farmland in Haerbin City, Northern of China. Red soil with a loamy clay texture was sampled from the farmland in Yingtan City, Southern of China. Alluvial soil with a silty clay texture was sampled from the farmland in Zhengzhou City, Middle East of China. Paddy soil with a silty clay loam texture was sampled from the farmland in Taizhou City, Southeast of China. At each location, soil was collected from a depth of 0–20 cm. Soil characteristics were determined, including soil texture, pH, organic matter, total N and total P for each of the four soil samples (Agricultural Chemistry Committee of China, 1983). The detail of the soil characteristics was shown in Supplementary Table S1. Soils were sterilized by autoclaving at 121°C for 15 min with intermittent incubation at 20°C for 2 days (Liu and Mustapha, 2014), as described in the Supplementary Text S1.

### Bacterial Strains and Cultivation Conditions

The strain used in this study was *E. coli* O157:H7 EDL933 (ATCC 43895). A single bacterial colony was initially inoculated into 10 mL of Luria-Bertani (LB) medium, and incubated at 37°C for 4 h with shaking at 150 rpm. Then a 1.0 mL of suspension was transferred to 250 mL of LB medium and incubated at 37°C for 10 h with agitation (150 rpm). Bacterial suspension of *E. coli* O157:H7 in late exponential phase was obtained. Bacteria were harvested by centrifugation at 8000 *g* for 10 min at 4°C, washed three times with 0.15 M phosphate buffered saline (PBS) and adjusted to OD_600_ = 1.0. Bacterial concentrations were quantified by spreading 100 μL of diluted samples on Tellurite Cefixime – Sorbitol MacConkey Agar (TC-SMAC, Fish Scientific, United States) plates for 24 h at 37°C, and counting the CFU.

### Preparation of Viable and Dead Cells

A series of concentrations of viable *E. coli* O157:H7 was obtained by serial dilutions. The bacterial suspensions were adjusted to cell concentrations between 10^3^ and 10^8^ CFU/mL. The suspension was divided into two aliquots. One aliquot was used as the viable sample, and the other aliquot was used to prepare dead samples. To obtain dead cells, suspension was heated in water bath at 75°C for 10 min. To ensure the death of cell, 10 μL of heated *E. coli* O157:H7 was plated on TC-SMAC plates and the same volume of *E. coli* O157:H7 without heat treatment on another plate as positive control. The plates were placed at 37°C and no colony growth was found after 24 h.

### Artificial Contamination of Soil Samples

To simulate a high ratio of dead cells in soils, we used sterilized soil inoculated with a mixture of viable and dead *E. coli* O157:H7 (viable/dead ratio of 0.001%). For the optimized PMA-qPCR assay, four agricultural soils were artificially contaminated with a mixture of viable and dead *E. coli* O157:H7 (viable/dead ratio of 0.001%), with a final concentrations of 10^2^ to 10^6^ viable cells/g in triplicate. To determine the fate of *E. coli* O157:H7 in paddy soil, *E. coli* O157:H7 were inoculated into 10 g paddy soil to a final concentration of 10^7^ CFU/g. Culturable number, viable number and total number of *E. coli* O157:H7 in paddy soil were quantified after incubation at 25°C during 40 days.

### Cell Extraction From Soil

Cells were separated from the soil matrix following the previous method (Macdonald, 1986; Burkert et al., 2019) with slight modification, as described in the Supplementary Material. To evaluate the recovery rate of *E. coli* O157:H7 separated from different soil debris, we extracted *E. coli* O157:H7 in a 10 g soil sample with 40 mL 0.15 M PBS, 0.1% peptone buffer and 0.85% NaCl, respectively. The detailed procures and results are described in the Supplementary Text S2. Culture-based methods were used to evaluate the effect of the cell extraction process on the recovery rate of *E. coli* O157:H7.

### Turbidity Measurement of Soil Suspension

The turbidity of soil suspensions was measured on Hach Turbidimeter 2100N (Hach, United States). The assay protocol was adapted from a previous study (Zhu et al., 2016). Briefly, 10 g of each soil was mixed with 100 mL of deionized water, and shaken for 12 h. To avoid excessive turbidity, the soil suspensions were diluted to six dilutions ranging from 75 to 2.5%. The soil suspensions were agitated and immediately placed into a turbidimeter cell. The turbidity unit is reported in NTU. The range of turbidity is 0–4000 NTU. The soil suspensions from four soils and cell suspensions extracted from soils were measured in triplicate.

### Centrifugation in Density Gradient

To reduce the proportion of dead bacteria, we used the density gradient centrifugation method (Makinoshima et al., 2002). The resuspended suspension was layered over 1 mL Percoll solutions in a 2 mL microcentrifuge tube. The microcentrifuge tubes were centrifuged at 13,000 *g* for 30 min at 4°C. We gathered the upper, middle and bottom phases containing cells into another 2 mL microcentrifuge tube. Viable cells suspension was obtained from the middle and bottom Percoll layer. The removal efficiency of dead bacteria and recovery of viable bacteria was evaluated by the four solutions; 100% Percoll (1.127 g/mL), 70% Percoll (1.090 g/mL), 50% Percoll (1.067 g/mL), and 30% Percoll (1.043 g/mL).

### Direct Viable Count and Live/Dead BacLight Dyeing

After cell extraction from the soil, direct viable count (DVC) was used to count viable bacterial cells, For DVC, triplicate bacterial suspensions were enriched with 0.025% (W/V) yeast extract (Difco, United States) and 0.002% (W/V) nalidixic acid (Sigma-Aldrich, United States), and incubated in darkness at 20°C. After incubation for 6 h, DVC were determined by the epifluorescent technique. The number of elongated or obesity and fluorescent red-orange granules was counted. To evaluate the distribution of dead and viable cells in different density layers, a Live/Dead BacLight Bacterial Viability Kit (Invitrogen, CA, United States) was used to differentially stain live and dead cells by fluorescence microscopy following the manufacturer’s protocol, detailed in Supplementary Text S3.

### PMA-qPCR

In all PMA-qPCR experiments, each bacterial sample contained 1 mL suspension of viable or dead cells. All samples were tested in triplicate. The assay protocol was adapted from a previous study (Li and Chen, 2012). Briefly, PMA (Biotium, Inc., CA, United States) was dissolved in dimethyl sulfoxide (Sigma-Aldrich)-water to attain a 20 mM stock solution and stored in the dark at −20°C. One milliliter of bacterial suspension containing viable cells, dead cells and mixture of viable and dead cells were put into light-transparent 1.5 mL micro-centrifuge tubes, respectively. A final concentration of 50 μM PMA was added in light-transparent 1.5 mL micro-centrifuge tubes. Identical volumes of 20% DMSO without PMA were used as controls. The cells were then subjected to 10 min dark incubation, and 15 min light exposure by a 650W halogen lamp (OSRAM, Germany). To minimize the effect of halogen lamp irradiation, the microcentrifuge-tubes were placed horizontally on ice. The distance between the centrifuge tube and the halogen lamp was up to 20 cm. During light exposure, the sample tubes were occasionally mixed. Finally, these tubes were centrifuged at 12,000 *g* for 10 min. The supernatant was removed carefully, and the pellets were collected for further DNA extraction. DNA was extracted using the Fast DNA SPIN kit for soil (MP Biomedicals; Santa Ana, CA, United States) according to the manufacturer’s protocol. The concentrations of the extracted DNA were measured by NanoDrop (Thermo Fisher Scientific, United States) and given in Supplementary Table S2.

The qPCR experiments were performed on LightCycle 480 Software Setup (Roche Diagnostics Ltd., Rotkreuz, Switzerland). For TaqMan assays for amplification of Z3276 gene, each 10 μL reaction volume contained 5 μL of 2 × Probe Premix (Takara, Dalian, China), 0.2 μM each primer, 0.35 μM probe, 0.1 μL ROX, and 1 μL DNA template. The qPCR conditions were as follows: activation of TaqMan probe at 95°C for 10 min, 40 cycles of denaturation at 95°C for 10 s and annealing at 60°C for 1 min. In addition, the genomic DNA of *E. coli* O157:H7 was used as a template for setting up the standard curve. Each reaction was amplified in triplicate. Negative controls were set in each qPCR reaction. The serially tenfold diluted bacterial concentrations (Log CFU) were plotted against threshold cycles (Ct) values. The standard curve of CFU-Ct was used to calculate the cell equivalents, mentioned in Supplementary Figure S8. The range of qPCR was 2.0 to 7.0 log CFU/reaction. In order to obtain the appropriate Ct values, the DNA template was 10-fold diluted.

### Optimization of Extractants on Recovery of *E. coli* O157:H7 From Soils

**FIGURE 1:**
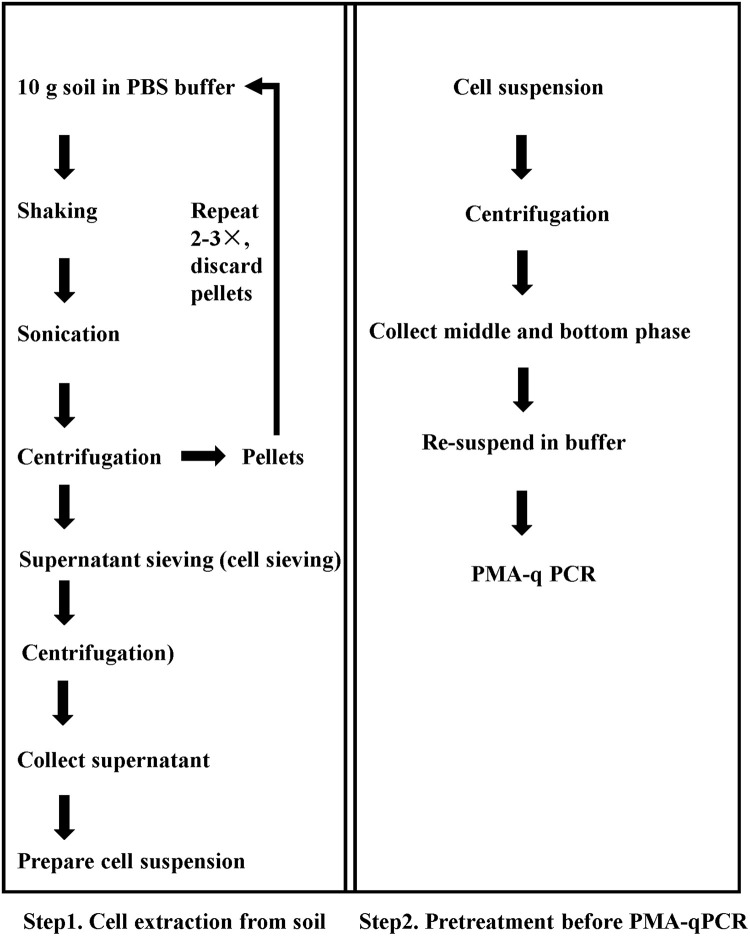
Sequential procedure for pretreatment for viable pathogen detection in soil matrix by the optimized PMA-qPCR.

To evaluate the effect of extractants on recovery of *E. coli* O157:H7 from soils, viable *E. coli* O157:H7 was inoculated into different soils to a final concentration of 10^5^ CFU/g. *E. coli* O157:H7 cells were extracted from soils with 0.15 M PBS, 0.1% peptone buffer and 0.85% NaCl, respectively. The detailed procures are present in Figure 1 and described in the Supplementary Material. Culture-based methods were used to evaluate the effect of the cell extraction process on the recovery rate of *E. coli* O157:H7.

### Optimization of PMA Treatment on Viable Cells

To determine the PMA toxic effect on viable *E. coli* O157:H7 cells after the optimized pretreatment, a suspension of viable cells (10^5^ CFU/mL) was treated with PMA separately at varying final concentrations of 25, 50, 75, and 100 μM. Viable cells without PMA treatment were used as controls.

### Optimization of the Method in the Presence of a High Ratio of Dead Cells

Agricultural soils harbor high proportion of dead cells, and there is a limitation of PMA deficiency in presence of high dead bacteria in soils. So *E. coli* O157:H7 was inoculated into 10 g paddy soil to final concentration of 10^2^ viable cells/g and 10^7^ dead cells/g. The number of viable *E. coli* O157:H7 was compared by the direct PMA-qPCR and the optimized PMA-qPCR. In addition, the same concentration of viable cells in water was set as positive control, paddy soil without *E. coli* O157:H7 inoculated and paddy soil inoculated heat-killed *E. coli* O157:H7 were used as negative controls. All samples were set in triplicate.

### Application of the Optimized PMA-qPCR to Agricultural Soils

The optimal conditions of the optimized PMA-qPCR were tested on four agricultural soils in China. Each soil sample was inoculated with serially 10-fold diluted viable cells and 10^5^ dead cells/g of *E. coli* O157:H7. All samples were set in triplicate. In order to validate the accuracy of the optimized PMA-qPCR, the differences in the number of viable cells were compared between the culture-based methods, DVC method and the optimized PMA-qPCR within the agricultural soils. In order to apply the optimized PMA-qPCR for the detection of VBNC *E. coli* O157:H7 in paddy soil, the fate of *E. coli* O157:H7 in paddy soil was studied. *E. coli* O157:H7 were inoculated into 10 g paddy soil to a final concentration of 10^7^ CFU/g. Culturable number, viable number and total number of *E. coli* O157:H7 in paddy soil were quantified after incubation at 25°C during 40 days.

### Statistical Analysis

In the optimization of PMA concentration on viable cells, data were analyzed by one-way ANOVA followed by a *post hoc* Tukey’s test. When comparing the data from the optimized PMA-qPCR with those from other methods, a two-tailed paired Student’s *t*-test was used, and *p* < 0.05 was set as the significant difference.

## Results and Discussion

A flow chart of the complete procedure is given in Figure 1. Step 1 was used for cell extraction and turbidity reduction, and Step 2 was used to reduce the proportion of dead bacteria. Soil samples with mixture of dead and viable cells were tested, and the Ct values were obtained from qPCR assays.

### Reduction the Interference From Soil Matrix

In agricultural soils, the high turbidity of soil suspension caused by soil particles and organic matter hinders the penetration of light, inhibits the cross-linking of PMA and DNA, and thus could adversely affects the availability of PMA (Luo et al., 2010). The number of NTU is one of the parameters to evaluate water turbidity levels. In this study, the results indicated that the turbidity of four soil suspensions decreased from 1000–3500 to 28.4–67.0 NTU after Step 1 (Supplementary Figure S1). In addition, Ct values were obtained from examination of viable *E. coli* O157:H7 cells. Under this turbidity condition, the Ct value obtained from viable *E. coli* O157:H7 is accurate compared to the positive control (Supplementary Figure S2). These results suggested that this method can effectively reduce the turbidity of the soil matrix, and successfully repress the signal of DNA derived from dead cells by PMA. The turbidity condition (28.4–67.0 NTU) is consistent with the reported turbidity condition of PMA, where PMA-qPCR can function properly in relatively high NTU conditions [<120 NTU (Yuan et al., 2018) or total suspended solids = 1000 mg/L (Bae and Wuertz, 2009)].

A high proportion of dead bacteria (or extracellular DNA) in the soil background could affect the efficiency of PMA. As a large number of dead cells may have higher dye absorption ability, resulting in fewer dye molecules available to bind with DNA (Fittipaldi et al., 2012). Therefore, we used gradient density centrifugation in the pretreatment step to reduce the interference of a high proportion of dead bacteria in the soil background. It was found that the recovery rate of *E. coli* O157:H7 has reached 100% in the high density layer from 50 to 30% Percoll. The recovery rate of *E. coli* O157:H7 was 35.4 ± 7.36% and 70.4 ± 9.45% in the high density layer from 70 to 100% Percoll. The results suggested that 50 and 30% Percoll did not affect the culturability of *E. coli* O157:H7 (Supplementary Figure S3).

Importantly, after 50% Percoll treatment, the number of total *E. coli* O157:H7 decreased by three orders of magnitude and could be excluded by subsequent PMA after pretreatment (Supplementary Figure S3). The results showed that the ratio of viable cells to dead cells increased from 0.001% to 1.025% (Supplementary Figure S3). This ratio of viable and dead cells was consistent with the reported condition of PMA, where the ratio of viable and dead cells could not be less than 0.1% (Fittipaldi et al., 2012), or the ratio of viable cells to dead cells of *E. coli* should be more than 1%, and the number of free DNA gene copies should not be more than 4 × 10^5^ (Kantonales Laboratorium, 2009). To our knowledge, this is the first report of applying density gradient centrifugation steps before PMA treatment to eliminate the interference of dead bacteria in complex environmental samples, aiming to be applied to various soil media in the presence of a high proportion of dead cells or extracellular DNA.

### Optimization of PMA Concentration

To establish a new procedure for PMA-qPCR, the concentration of PMA was optimized. The results showed that 100 μM PMA was toxic to 10^5^ CFU/mL *E. coli* O157:H7, resulting in false negative results (Supplementary Figure S4). Previously it has been reported that higher concentration of PMA posed toxic effects to bacteria (Nocker et al., 2006). In addition, 50 μM PMA completely inhibited the amplification of 10^7^ dead cells/mL. To effective exclusion of DNA signals from dead cell, the concentration of PMA used in this study was consistent with previous study in fresh-cut vegetables (Elizaquivel et al., 2012). However, increased PMA concentration (100 μM) was used for viable pathogens quantification in complex environmental samples (Li D. et al., 2014; Ruike et al., 2016). In this study, 50 μM PMA was suitable for the detection of viable *E. coli* O157:H7 in different soils by the optimized PMA-qPCR (Supplementary Figure S4).

### Validation of the Optimized PMA-qPCR Assay

**FIGURE 2:**
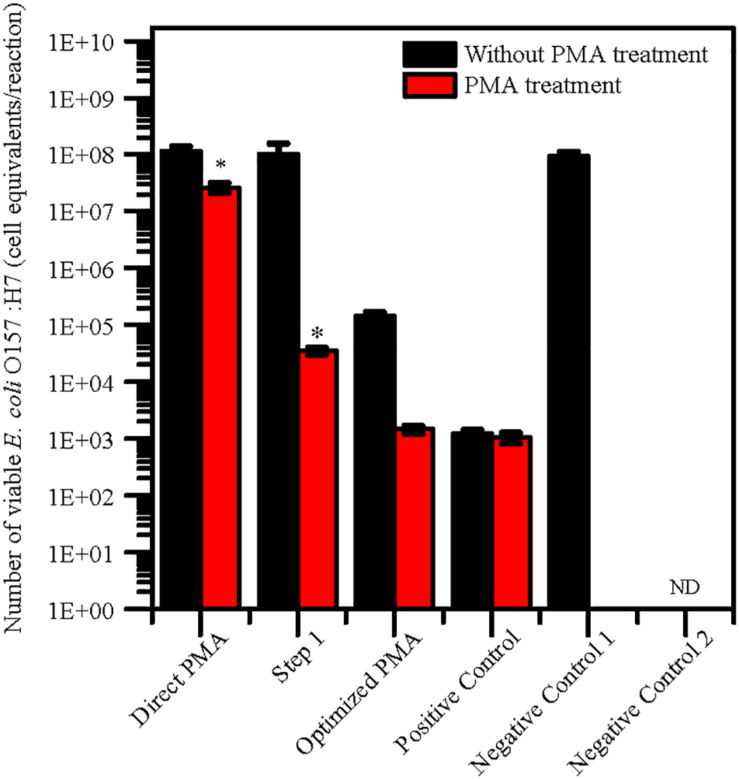
Application of PMA treatment after pretreatment for 10 g paddy soils inoculated with 10^2^ viable cells/g and 10^7^ dead cells/g of *E. coli* O157:H7 cells. The same concentration of viable *E. coli* O157:H7 cells inoculated in water were set as positive control. PMA-qPCR and qPCR results indicated mean values (*n* = 3) of the pathogen concentrations. Error bars represent the standard deviations of the mean (SD). “Direct PMA” represent soil samples without pretreatment. “Step 1” represent soil samples with pretreatment after Step 1 procedure. “Optimized PMA” represent soil samples with the optimized pretreatment. “Negative Control 1” and “Negative Control 2” represent soil samples inoculated with heat-killed *E. coli* O157:H7 and without *E. coli* O157:H7. “ND” represent “not detected.” The number of viable *E. coli* O157:H7 by PMA treatment was compared with positive control group by the two-tailed paired Student’s *t*-test was used (*p < 0.05).

The levels of quantification capacity of both the optimized PMA-qPCR and direct PMA-qPCR were evaluated and compared in mixture of viable and dead cells. *E. coli* O157:H7 was inoculated into 10 g paddy soil to final concentration of 10^2^ viable cells/g and 10^7^ dead cells/g. The cell equivalents of *E. coli* O157:H7 was used for validation of the optimized PMA-qPCR. The results exhibited that significantly higher numbers of *E. coli* O157:H7 by direct PMA-qPCR method compared with positive control. However, the number of viable *E. coli* O157:H7 detected by the optimized method and the positive control was consistent (Figure 2). In comparison, the optimized PMA-qPCR method could accurately detect 10^2^ CFU/g of viable cells in the presence of a large number of dead cells (10^7^ dead cells/g) in soils. The results suggested that the optimized PMA-qPCR method is more effective methods as compared to other methods for detection of viable cells in the presence of a large number of dead cells.

### Application of the PMA-qPCR Method to Agricultural Soils

**FIGURE 3:**
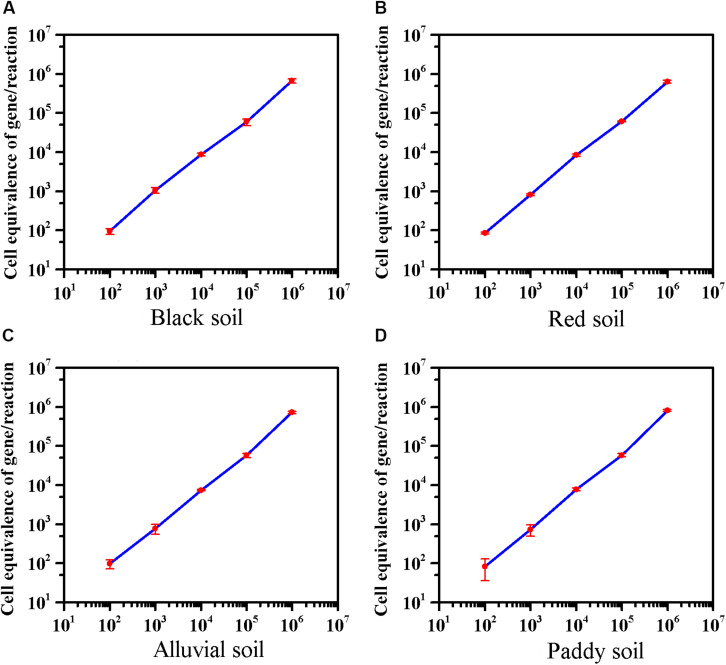
The sensitivity of the optimized PMA-qPCR for detection of viable *E. coli* O157:H7 in the presence of dead cells in four soils. The abscissa represents the theoretical dosage of *E. coli* O157:H7 (Culture-based method), and the ordinate represents the cell equivalents of gene Z3276 detected by the optimized PMA-qPCR method. **(A)** Black soil; **(B)** red soil; **(C)** alluvial soil; **(D)** paddy soil. PMA-qPCR results showed the mean concentrations (*n* = 3) of the viable *E. coli* O157:H7. Error bars represent the standard deviations of the mean (SD).

The effect of extractants on recovery of *E. coli* O157:H7 from the different soils was evaluated. In black soil and red soil, the extractants on recovery of *E. coli* O157:H7 were as follows: PBS, Peptone > NaCl. Furthermore, the recovery of *E. coli* O157:H7 by PBS was significantly higher than NaCl in alluvial soil. The extractants on recovery of *E. coli* O157:H7 in paddy soil were as follows: PBS > NaCl > Peptone (Supplementary Figure S5). As low as 8–10 cells of *E. coli* O157:H7 can cause disease (Herold et al., 2004), 100 CFU/10 g of *E. coli* O157:H7 was inoculated into soils. The average detection value was 95 in black soil, 86 in red soil, 98 in alluvial soil, and 83 in paddy soil. The method can detect *E. coli* O157:H7 in four soils with a sensitivity of 10 CFU/g (Figure 3). The sensitivity of viable *E. coli* O157:H7 in soils by this method was an order of magnitude lower than the previous reports on PMA-qPCR without enrichment (Gyawali et al., 2016; Casanovas-Massana et al., 2018).

To further determine the accuracy of this method, it was compared with traditional culture methods and direct microscopy method. The number of *E. coli* O157:H7 in soils determined by PMA-qPCR and culture methods were similar (Supplementary Figure S6). The result was in agreement with another study (Shah et al., 2019), where spiked *Salmonella Newport* densities in soils determined by PMA-qPCR and culture methods were similar. The optimized PMA-qPCR assay was completed with considerable economy of time (6 h versus 24 h) and achieved accurate quantitative capacity comparable to culture assay. In addition, direct microscopy is considered to be a reliable method for counting viable bacteria, and is widely used for soil (Jjemba et al., 2006; Troxler et al., 2012; Moreno-Mesonero et al., 2016). In this study, the optimized PMA-qPCR for viable *E. coli* O157:H7 was comparable to that of the direct viable count method (Supplementary Figure S7). These results suggested that the optimized PMA-qPCR may have a potential alternative to detect viable *E. coli* O157:H7 by microscopy and culture-based method in paddy soils, regardless of high turbidity and a high proportion of dead cells.

### The Survival of *E. coli* O157:H7 in Paddy Soils

**FIGURE 4:**
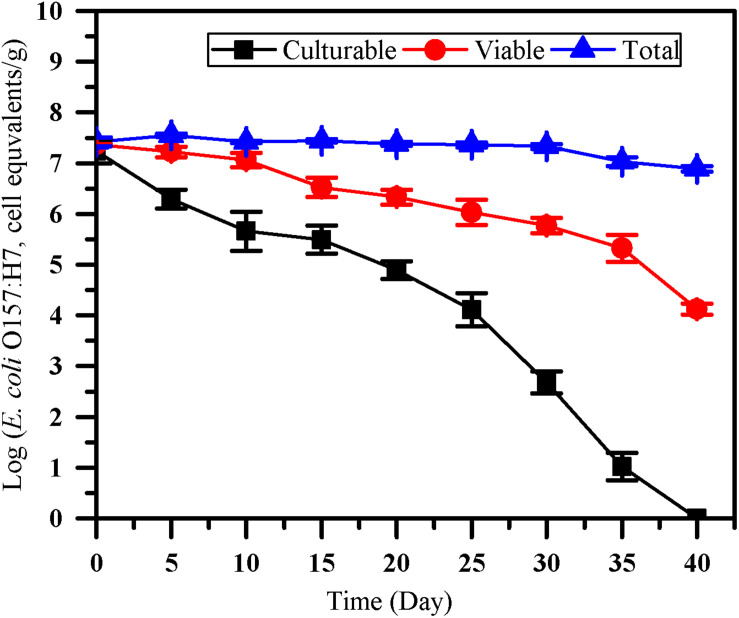
The survival of *E. coli* O157:H7 in paddy soil. Culturable number, viable number and total number of *E. coli* O157:H7 was measured by culture-based method (squares), the optimized PMA-qPCR (circles), and qPCR (triangles), respectively. Error bars indicate standard deviations.

The number of culturable, viable and total cells of *E. coli* O157:H7 was monitored by culture method, the optimized PMA-qPCR and qPCR (Figure 4). After inoculated into paddy soil for 40 days, no culturable *E. coli* O157:H7 cells were detected by the culture method (<0.1 CFU/g). However, VBNC cell count of 4.13 ± 0.11 log cell equivalents/g were detected after 40 days of incubation in paddy soils. The results showed that the difference between viable and culturable cell counts increased over time, indicating that the accumulation of VBNC *E. coli* O157:H7 may occur on prolonged exposure in the paddy soils. PMA-qPCR method was widely used for quantifying VBNC *E. coli* O157:H7 in fresh produce (Dinu and Bach, 2013; Han et al., 2020). However, to our knowledge, there is limited study on quantification of *E. coli* O157:H7 in the VBNC state by PMA-qPCR in soils. As VBNC state cells are still alive and maintain the integrity of membrane, it is accessible to further apply the optimized PMA-qPCR for the detection of *E. coli* O157:H7 in the VBNC state after their completely loss of culturability as confirmed by culture methods.

Although the optimized PMA-qPCR assay has shown great success in detecting viable *E. coli* O157:H7 in agricultural soil, it still needs to be verified in more soil types due to the complexity of the soil. In addition, the low efficiency of extracting cells from the soil may result in DNA loss (Williamson et al., 2011), and the extraction process will be further optimized.

## Conclusion

We developed a rapid, sensitive and accessible PMA-qPCR for detection and quantification viable *E. coli* O157:H7 in agricultural soil. Coupled with a culture method, the PMA-qPCR could quantify *E. coli* O157:H7 in the VBNC state in the samples of agricultural soils, which evade detection by conventional culture-based methods. Significant economies of time (6 h versus 24 h) and sensitivity (100 CFU/10 g soil) were found in comparison with culture-based method. This method could comprehensively evaluate the survival of *E. coli* O157:H7 in the agricultural soils. In addition, we focused on optimizing the limiting factors of the method applied to the soils, turbidity, including high proportion of dead bacteria and PMA concentration. The maximum removal of DNA from dead cells was achieved by 1.067 g/mL Percoll of centrifugation and 50 μM PMA treatment. Our study validated the performance of PMA-qPCR for the detection and quantification viable *E. coli* O157:H7 in the agricultural soils, and improved the applicability of this method. The optimized PMA-qPCR could be further used to identify and quantify sources of contamination in time and eventually reduce the prevalence of viable *E. coli* O157:H7 in agricultural soil.

## Data Availability Statement

All datasets generated for this study are included in the article/Supplementary Material.

## Author Contributions

YF contributed to the experimental design and preparation of the first draft of the manuscript. ZY contributed to the data analysis. YJ and JF conducted part of the experiments. MH improved English language and helped in revision. CS contributed to the experimental design, manuscript editing, and the final version of the manuscript. All authors contributed to the article and approved the submitted version.

## Conflict of Interest

The authors declare that the research was conducted in the absence of any commercial or financial relationships that could be construed as a potential conflict of interest.
