# Supplementary material for: An Optimized Method to Assess Viable Escherichia coli O157:H7 in Agricultural Soil Using Combined Propidium Monoazide Staining and Quantitative PCR
Source: Front Microbiol. 2020 Jul 31;11:1809. doi: 10.3389/fmicb.2020.01809 (PMC7411311; doi:10.3389/fmicb.2020.01809)
Supplement: Supplementary file 1 [file Table_1.DOC]

Supplementary Material

**Supplementary Material and Method:**

**Text S1.** The procedure of sterilized soils

**Text S2.** Cell extraction from soils

**Text S3.** Direct viable count (DVC) and Live/Dead BacLight dyeing

**Supplementary Figures:**

**Figure S1.** Turbidity change after soils pretreatment.

**Figure S2.** Application of PMA treatment on viable *E. coli* O157:H7 detection in different soils after pretreatment.

**Figure S3.** (A). Ratio of culturable *E. coli* O157:H7 in low density band and high density band of gradient density. (B). Quantification of *E. coli* O157:H7 by qPCR in high density band of 30% and 50% gradient density. (C) The ratio of viable cells to dead cells in high density band of 30% and 50% gradient density. (D). Quantification of *E. coli* O157:H7 by PMA-qPCR in high density band of 30% and 50% gradient density.

**Figure S4.** (A). Effect of PMA concentration on 10^5^ CFU/reaction viable cells of *E. coli* O157:H7. (B). Effect of PMA concentration on 10^5^ CFU/reaction viable cells 10^7^ CFU/reaction dead cells of *E. coli* O157:H7. (C). Application of 50 mM PMA on discrimination viable cells and dead cells of *E. coli* O157:H7 in different soils.

**Figure S5.** The recovery of *E. coli* O157:H7 by different extractants.

**Figure S6.** Comparison the optimized method with culture-based method by spiking mixture of viable and dead *E. coli* O157:H7 to four soils.

**Figure S7.** Comparison the optimized method with direct microscopy method by spiking mixture of viable and dead *E. coli* O157:H7 to four kinds of soils.

**Figure S8.** The standard curve of Ct-log CFU.

**Supplementary Tables**

**Table S1.** Basic physio-chemical characteristics of studied soils.

**Table S2.** Concentrations of extracted DNA.

**Text S1. The procedure of sterilized soils**

In brief, different soils samples were air-dried, passed through a 2 mm pore-size sieve, well mixed, dried in an oven at 105 ℃ for 24 h and sterilized 3 times by autoclaving at 121 ℃ for 15 min with intermittent incubation at 20 ℃ for 2 days. Sterility was confirmed by plating soil suspension on R_2_A agar (R_2_A, Difco Laboratories, USA) for 10 days. The moisture contents of soils were adjusted by adding sterile deionized water until field capacity (soil water content at -33 kPa). Soil texture, pH, organic matter, total N and total P for each of the four soil samples were analyzed (Agricultural Chemistry Committee of China, 1983).

**Text S2. Cell extraction from soils**

After sonication for 1 min at 20V using QSonica ultrasonicator on ice, sonicated samples were centrifuged at 500 g for 10 min at 4 ℃ to remove large particles and debris. The pellets were re-suspended in PBS and washed 2-3 times, then pellets were discarded following the procedure (Fig. 1). The supernatant was sieved by cell sieve (100 μm) and gathered with centrifugation at 8, 000 g for 10 min at 4 ℃ and re-suspended in 0.2 mL 0.15 M PBS. The re-suspended suspension was extracted and layered it over 1 mL 70% Percoll (1.090 g/mL) solutions in a 2 mL microcentrifuge tubes. The microcentrifuge tubes were centrifuged at 13, 000 g for 30 min at 4 ℃ to remove clay particles. The upper and middle phase containing cells were transferred into another 2 mL microcentrifuge tube and centrifuged at 8, 000 g for 10 min at 4 ℃. The supernatant was discarded, and the pellets was re-suspended in 200 μL of 0.15 M PBS solution. Culture-based method was used to evaluate the effect of cell extraction process on recovery rate of *E. coli* O157:H7.

**Text S3. Direct viable count (DVC) and Live/Dead BacLight dyeing**

For DVC, triplicate bacterial suspension was enriched with 0.025% (W/V) yeast extract (Difco, USA) and 0.002 % (W/V) nalidixic acid (Sigma, USA), and incubated in darkness at 20 ℃. After incubation for 6 h, DVC were determined by the epifluorescent technique. The number of elongated or obesity and fluorescent red-orange granules was counted.

For Live/Dead BacLight dyeing, Live/Dead BacLight Bacterial Viability Kit (Invitrogen Detection Technoligies, CA) was used to differentially stain live and dead cells by fluorescence microscope followed as the manufacturer’s protocol. Briefly, 3 μL of 1:1 mixture of 3.34 mM SYTO 9 and propidium iodide (PI) solutions were added to 500 μL suspensions. Suspensions were stained for 15 min at 25 ℃.

**Figure S1.** Turbidity change of soil suspension after pretreatment (1 g soil in 9 mL phosphate buffered saline). Differences of turbidity between pretreatment and without pretreatment were compared by the two-tailed paired Student’s t-test was used (*, p ＜ 0.05). Turbidity results indicated mean values (n=3) of the samples. Error bars represent the standard deviations of the mean (SD).

**Figure S2.** Application of PMA treatment after pretreatment for soils inoculated with 10^5^ viable cells/g s and 10^7^ dead cells/g of *E. coli* O157:H7. The same concentration of viable and dead *E. coli* O157:H7 cells inoculated in water were set as control. Threshold cycles (Ct) results indicated mean values (n=3) of the pathogen concentrations. Error bars represent the standard deviations of the mean (SD). Differences of Ct values between control and four soils were compared by the two-tailed paired Student’s t-test was used (*, p＜0.05).

**Figure S3**. The distribution of 10^3^ viable cells/mL and 10^8^ dead cells/mL of *E. coli* O157:H7 in different gradient density. (A). Ratio of culturable *E. coli* O157:H7 in low density band and high density band of gradient density. (B). Quantification of *E. coli* O157:H7 by qPCR in high density band of 30% and 50% Percoll gradient density by qPCR. (C) The ratio of viable cells to dead cells in high density band of 30% and 50% gradient density. (D). Quantification of *E. coli* O157:H7 by PMA-qPCR in high density band of 30% and 50% Percoll gradient density. The same concentration of total (B) and viable (D) *E. coli* O157:H7 inoculated in water were set as controls. The number of *E. coli* O157:H7 in the Percoll density group was compared with that in the control group by the two-tailed paired Student’s t-test was used (*, p ＜ 0.05). “Untreated” represent samples without gradient density centrifugation pretreatment. “ND” indicates “Not detected”. Gene copies results indicated mean values (n=3) of the samples. Error bars represent the standard deviations of the mean (SD).

**Figure S4.** (A). Effect of PMA concentration on 10^5^ viable cells /mL of *E. coli* O157:H7. (B). Effect of PMA concentration on inhibition of PCR amplification of DNA from 10^5^ viable cells/mL and 10^7^ dead cells/mL of *E. coli* O157:H7. (C). Application of 50 μM PMA on discrimination viable cells and dead cells of *E. coli* O157:H7 in different soils. The same concentration of viable and dead *E. coli* O157:H7 inoculated in water was set as positive control. PMA-qPCR results indicated mean values (n=3) of the samples. Error bars represent the standard deviations of the mean (SD). Differences of threshold cycles (Ct) of *E. coli* O157:H7 between control and different treatment were compared by one-way ANOVA Student’s t-test (*, p ＜ 0.05). Differences of threshold cycles (Ct) of *E. coli* O157:H7 between control and different PMA concentrations were compared by one-way ANOVA Student’s t-test (*, p ＜ 0.05).

**Figure S5.** The influence of extractants on recovery of *E. coli* O157:H7 from soils inoculated with 10^5^ CFU/g. Data were analyzed by one-way ANOVA followed by a post hoc Tukey’s test. The same concentration of viable *E. coli* O157:H7 inoculated in water was set as positive control. Different letters indicate significant differences (p ＜ 0.05). Culturing results indicated mean values (n=3) of the samples. Error bars represent the standard deviations of the mean (SD).

**Fig. S6.** Comparison the optimized method with culture-based method by spiking mixture of viable and dead *E. coli* O157:H7 to four soils. The abscissa represents the theoretical dosage of *E. coli* O157:H7, ranging from 10^2^ to 10^6^ CFU/g. Differences of concentrations of viable *E. coli* O157:H7 between PMA-qPCR method and culture method were compared by one-way ANOVA Student’s t-test (*, p ＜ 0.05). PMA-qPCR results indicated mean values (n=3) of the samples. Error bars represent the standard deviations of the mean (SD).

**Figure S7.** Comparison the optimized method with direct microscopy method by spiking mixture of viable and dead *E. coli* O157:H7 to four soils. The abscissa represents the theoretical dosage of *E. coli* O157:H7. Differences of concentrations of viable *E. coli* O157:H7 between PMA-qPCR method and direct viable count method were compared by one-way ANOVA Student’s t-test (*, p ＜ 0.05). PMA-qPCR results indicated mean values (n=3) of the samples. Error bars represent the standard deviations of the mean (SD).

**
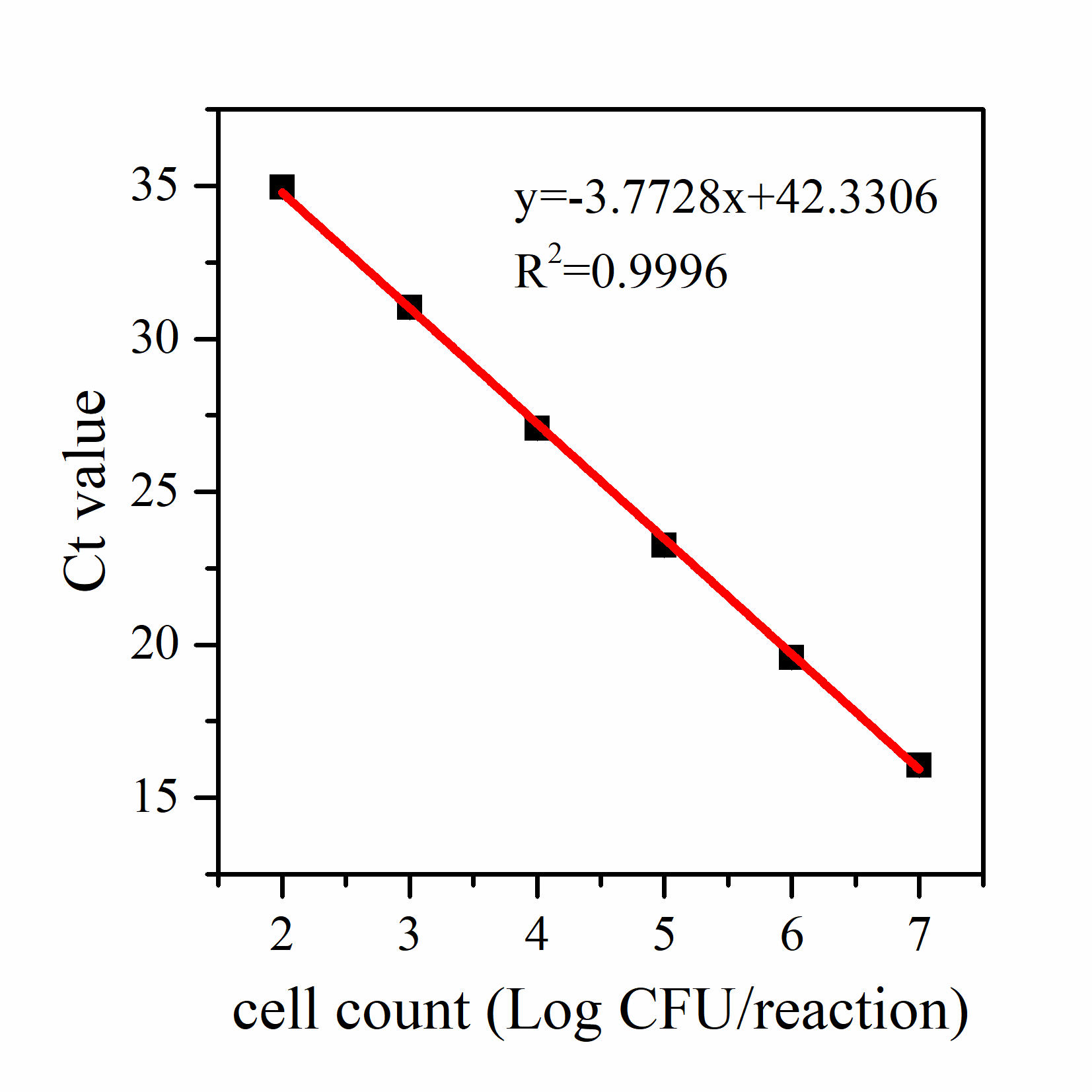
**

**Figure S8.** The standard curve of Ct-log CFU. The standard curve was produced from 10-fold dilutions of the *E. coli* O157:H7 ranging from 2.0 to 7.0 log CFU/reaction. Error bars represent the standard deviations of the mean (SD). The threshold cycles (Ct) were obtained from qPCR assays. Colony forming unit (CFU) were quantified by culture method.

**Table S1.** Basic physio-chemical characteristics of studied soils.

| Soil | Soil type | *P*_tot_  （mg g^-1^ dry soil） | *N*_tot_  （mg g^-1^ dry soil） | Organic matter  （mg g^-1^ dry soil） | pH | Clay content  (%) |
| --- | --- | --- | --- | --- | --- | --- |
| Paddy soil | Silty clay loam | 0.50 ± 0.04 | 3.01± 0.33 | 54.33± 5.32 | 6.61± 0.02 | 28.31 |
| Black soil | Silt clay | 0.63± 0.10 | 1.75± 0.14 | 33.30± 2.68 | 7.92± 0.07 | 36.18 |
| Alluvial soil | Silt loam | 0.82 ± 0.09 | 0.75± 0.18 | 12.18± 3.19 | 8.35± 0.05 | 11.09 |
| Red soil | Loamy clay | 0.89± 0.16 | 1.31± 0.25 | 22.57± 1.05 | 5.27± 0.02 | 38.52 |

**Table S2. The concentrations of extracted DNA**

| Sample NO. | Sample type | DNA Concentration (ng/μL) | DNA Concentration (μg/g) | OD260/280 |
| --- | --- | --- | --- | --- |
| BS1-1 | Black soil | 2.8 | 7.0 | 1.88 |
| BS1-2 | Black soil | 3.1 | 7.75 | 1.99 |
| BS1-3 | Black soil | 3.2 | 8 | 1.86 |
| BS2-1 | Black soil | 0.4 | 1 | 1.78 |
| BS2-2 | Black soil | 0.35 | 0.875 | 1.85 |
| BS2-3 | Black soil | 0.33 | 0.825 | 1.84 |
| RS1-1 | Red soil | 4.5 | 11.25 | 1.81 |
| RS1-2 | Red soil | 5.3 | 13.25 | 2.15 |
| RS1-3 | Red soil | 5.4 | 13.5 | 1.89 |
| RS2-1 | Red soil | 0.6 | 1.5 | 1.77 |
| RS2-2 | Red soil | 0.7 | 1.75 | 1.82 |
| RS2-3 | Red soil | 0.6 | 1.5 | 1.85 |
| AS1-1 | Alluvial soil | 12.5 | 31.25 | 1.88 |
| AS1-2 | Alluvial soil | 12.6 | 31.5 | 2.01 |
| AS1-3 | Alluvial soil | 16.8 | 42 | 1.96 |
| AS2-1 | Alluvial soil | 1.8 | 4.5 | 1.91 |
| AS2-2 | Alluvial soil | 1.5 | 3.75 | 1.78 |
| AS2-3 | Alluvial soil | 1.5 | 3.75 | 1.82 |
| PS1-1 | Paddy soil | 7.6 | 19 | 1.91 |
| PS1-2 | Paddy soil | 8.2 | 20.5 | 1.85 |
| PS1-3 | Paddy soil | 8.6 | 21.5 | 1.96 |
| PS2-1 | Paddy soil | 1.8 | 4.5 | 1.92 |
| PS2-2 | Paddy soil | 2.2 | 5.5 | 1.78 |
| PS2-3 | Paddy soil | 1.9 | 4.75 | 1.85 |
| PS2019-1 | Paddy soil | 145 | 362.5 | 1.88 |
| PS2019-1 | Paddy soil | 110 | 275 | 1.87 |
| PS2019-3 | Paddy soil | 128 | 320 | 1.90 |
| PS2019-PMA-1 | Paddy soil | 106 | 265 | 1.81 |
| PS2019-PMA-2 | Paddy soil | 115 | 287.5 | 1.77 |
| PS2019-PMA-3 | Paddy soil | 121 | 302.5 | 1.74 |
| PS2019-Wash-1 | Paddy soil | 60 | 150 | 1.84 |
| PS2019-Wash-2 | Paddy soil | 45 | 112.5 | 1.92 |
| PS2019-Wash-3 | Paddy soil | 39 | 97.5 | 1.86 |
| PS2019-Wash-PMA-1 | Paddy soil | 0.8 | 2 | 1.77 |
| PS2019-Wash-PMA-2 | Paddy soil | 0.6 | 1.5 | 1.68 |
| PS2019-Wash-PMA-3 | Paddy soil | 0.3 | 0.75 | 1.79 |
| PS2019-OPT-1 | Paddy soil | ND | ND | 1.85 |
| PS2019-OPT-2 | Paddy soil | ND | ND | 1.89 |
| PS2019-OPT-3 | Paddy soil | ND | ND | 1.86 |
| PS2019-OPT-PMA-1 | Paddy soil | ND | ND | 1.77 |
| PS2019-OPT-PMA-2 | Paddy soil | ND | ND | 1.68 |
| PS2019-OPT-PMA-3 | Paddy soil | ND | ND | 1.79 |
| PS-SUR-D0-1 | Paddy soil | 8.6 | 21.5 | 1.88 |
| PS-SUR-D0-2 | Paddy soil | 7.9 | 19.75 | 1.89 |
| PS-SUR-D0-3 | Paddy soil | 8.2 | 20.5 | 1.88 |
| PS-SUR-D0-PMA-1 | Paddy soil | 7.9 | 19.75 | 1.84 |
| PS-SUR-D0-PMA-2 | Paddy soil | 7.8 | 19.5 | 1.82 |
| PS-SUR-D0-PMA-3 | Paddy soil | 8.1 | 20.25 | 1.77 |
| PS-SUR-D5-1 | Paddy soil | 8.6 | 21.5 | 1.87 |
| PS-SUR-D5-2 | Paddy soil | 8.8 | 22 | 1.82 |
| PS-SUR-D5-3 | Paddy soil | 8.9 | 22.25 | 1.84 |
| PS-SUR-D5-PMA-1 | Paddy soil | 8.2 | 20.5 | 1.77 |
| PS-SUR-D5-PMA-2 | Paddy soil | 8.3 | 20.75 | 1.78 |
| PS-SUR-D5-PMA-3 | Paddy soil | 8.8 | 22 | 1.81 |
| PS-SUR-D10-1 | Paddy soil | 8.2 | 20.5 | 1.88 |
| PS-SUR-D10-2 | Paddy soil | 8.0 | 20 | 1.85 |
| PS-SUR-D10-3 | Paddy soil | 7.9 | 19.75 | 1.82 |
| PS-SUR-D10-PMA-1 | Paddy soil | 7.9 | 19.75 | 1.74 |
| PS-SUR-D10-PMA-2 | Paddy soil | 7.6 | 19 | 1.72 |
| PS-SUR-D10-PMA-3 | Paddy soil | 8.8 | 22 | 1.64 |
| PS-SUR-D15-1 | Paddy soil | 4.2 | 10.5 | 1.84 |
| PS-SUR-D15-2 | Paddy soil | 2.6 | 6.5 | 1.85 |
| PS-SUR-D15-3 | Paddy soil | 3.5 | 8.75 | 1.88 |
| PS-SUR-D15-PMA-1 | Paddy soil | 4.1 | 10.25 | 1.71 |
| PS-SUR-D15-PMA-2 | Paddy soil | 2.8 | 7 | 1.69 |
| PS-SUR-D15-PMA-3 | Paddy soil | 3.2 | 8 | 1.55 |
| PS-SUR-D20-1 | Paddy soil | 1.5 | 3.75 | 1.85 |
| PS-SUR-D20-2 | Paddy soil | 1.9 | 4.75 | 1.88 |
| PS-SUR-D20-3 | Paddy soil | 1.8 | 4.5 | 1.88 |
| PS-SUR-D20-PMA-1 | Paddy soil | 1.2 | 3 | 1.65 |
| PS-SUR-D20-PMA-2 | Paddy soil | 0.9 | 2.25 | 1.64 |
| PS-SUR-D20-PMA-3 | Paddy soil | 1.1 | 2.75 | 1.52 |
| PS-SUR-D25-1 | Paddy soil | 0.4 | 1 | 1.87 |
| PS-SUR-D25-2 | Paddy soil | 0.3 | 0.75 | 1.88 |
| PS-SUR-D25-3 | Paddy soil | 0.4 | 1 | 1.88 |
| PS-SUR-D25-PMA-1 | Paddy soil | 0.6 | 1.5 | 1.54 |
| PS-SUR-D25-PMA-2 | Paddy soil | 0.7 | 1.75 | 1.59 |
| PS-SUR-D25-PMA-3 | Paddy soil | 0.3 | 0.75 | 1.54 |
| PS-SUR-D30-1 | Paddy soil | 0.3 | 0.75 | 1.89 |
| PS-SUR-D30-2 | Paddy soil | 0.4 | 1 | 1.85 |
| PS-SUR-D30-3 | Paddy soil | 0.4 | 1 | 1.88 |
| PS-SUR-D30-PMA-1 | Paddy soil | 0.6 | 1.5 | 1.56 |
| PS-SUR-D30-PMA-2 | Paddy soil | 0.3 | 0.75 | 1.70 |
| PS-SUR-D30-PMA-3 | Paddy soil | 0.2 | 0.5 | 1.53 |

BS, RS, AS and PS present black soil, red soil, alluvial soil and paddy soil. “PMA” present different samples after PMA treatment. “ND” stood for “Not detected”.
